# Supplementary material for: A non-canonical mechanism for Crm1-export cargo complex assembly
Source: eLife. 2015 Apr 21;4:e05745. doi: 10.7554/eLife.05745 (PMC4402694; doi:10.7554/eLife.05745)
Supplement: Supplementary file 1. — Yeast strains used in this study. DOI: http://dx.doi.org/10.7554/eLife.05745.013 [file elife05745s001.docx]

**Supplementary Table 1.** Yeast strains used in this study

| **Strain name** | **Genotype** | **Origin** |
| --- | --- | --- |
| *ENP1-GFP* | *MATa ENP1-GFP::HIS3MX ura3 leu2 met15 TRP1* | Open biosystems |
| *ENP1-TAP slx9∆* | *MATa Enp1-TAP::HIS3MX slx9::KANMX ura3 leu2 met15 TRP1* | Faza *et al*., 2012 |
| *GAR1-GFP* | *MATa GAR1-GFP::HIS3MX ura3 leu2 met15 TRP1* | Open biosystems |
| *GAR1-mCherry* | *MATa GAR1-mCherry::KANMX his3 leu2 met15 ura3* | Gift from Yves Barrals laboratory |
| *kar1-1 NUP82-mCherry* | *MATα kar1-1 NUP82-mCherry::KANMX ura3 leu2 ade1* | this study |
| *MEX67 shuffle* P*_gal1_-RIO2* | *MATa* P*_gal1_-RIO2::NATNT2 mex67::HIS3MX trp1 leu2* pRS316-*MEX67* | this study |
| *MEX67 shuffle slx9∆* | *MATa mex67::HIS3MX slx9::KANMX leu2 trp1* pRS316-*MEX67* | Faza *et al.*, 2012 |
| *MTR2 shuffle* P*_gal1_-RIO2* | *MATa* P*_gal1_-RIO2::NATNT2 mtr2::HIS3MX trp1 leu2* pRS316-*MTR2* | this study |
| *MTR2 shuffle slx9∆* | *MATa mtr2::HISMX slx9::KANMX leu2 trp1* pRS316-*MTR2* | Faza *et al.*, 2012 |
| *RIO2 shuffle* | *MATa rio2::NATNT2 his3 leu2 TRP1* pRS316-*RIO2* | this study |
| *RIO2 shuffle* | *MATa rio2::KANMX his3 leu2 TRP1* pRS316-*RIO2* | Schäfer *et al.*, 2002 |
| *RIO2 shuffle slx9∆* | *MATa rio2::NAT slx9::KANMX his3 leu2 TRP1* pRS316-*RIO2* | this study |
| *RIO2 shuffle yrb2∆* | *MATa rio2::NAT yrb2::KANMX his3 leu2 TRP1* pRS316-*RIO2* | this study |
| *rrp12-GFP slx9∆* | *MATa rrp12-GFP::HIS3MX slx9::NATNT2 ura2 leu2 TRP1* | this study |
| *SLX9-GFP* | *MATa SLX9-GFP::HIS3MX ura3 leu2 met15 TRP1* | Faza *et al.*, 2012 |
| *slx9∆* | *MATα slx9::KANMX ura3 his3 leu2 TRP1* | Faza *et al.*, 2012 |
| *yrb2∆* | *MATa his3 leu2 met15 ura3 yrb2::KANMX* | Open biosystems |
| *SLX9 shuffle yrb2∆* | *slx9::NATNT2 yrb2::KANMX leu2 his3 TRP pRS316-SLX9* | this study |
