# Supplementary material for: A non-canonical mechanism for Crm1-export cargo complex assembly
Source: eLife. 2015 Apr 21;4:e05745. doi: 10.7554/eLife.05745 (PMC4402694; doi:10.7554/eLife.05745)
Supplement: Supplementary file 2. — Plasmids used in this study. DOI: http://dx.doi.org/10.7554/eLife.05745.014 [file elife05745s002.docx]

**Supplementary Table 2.** Plasmids used in this study

| **Plasmid name** | **Gene** | **Origin** |
| --- | --- | --- |
| pEM1-*SLX9* | *AMP^R^ JB1-HIS_6_-SLX9* | this study |
| pEM1-*slx9-1* | *AMP^R^ JB1-HIS_6_-slx9-1* | this study |
| pET47b-*NMD3* | *KAN^R^ HIS_6_-NMD3* | this study |
| pGEX-4TEV-*KAP123* | *AMP^R^ GST-KAP123* | Fries *et al.*, 2007 |
| pGEX-6P-1-*LTV1* | *AMP^R^ GST-LTV1* | this study |
| pGEX-6P-1-*LTV1∆NES* | *AMP^R^ GST-LTV1∆NES* | this study |
| pGEX-6P-1-*NTF2* | *AMP^R^ GST-NTF2* | this study |
| pGEX-6P-1-*RIO2* | *AMP^R^ GST-RIO2* | this study |
| pGEX-6P-1-*RIO2∆NES* | *AMP^R^ GST-RIO2∆NES* | this study |
| pGEX-6P-1-*RIO2-NMD3NES* | *AMP^R^ GST-RIO2-NMD3NES* | this study |
| pGEX-6P-1-*RIO2^3G^* | *AMP^R^ GST-RIO2^3G^* | this study |
| pGEX-6P-1-*RIO2^AG^* | *AMP^R^ GST-RIO2^AG^* | this study |
| pGEX-6P-1-*SLX9* | *AMP^R^ GST-SLX9* | this study |
| pGEX-6P-1-*slx9-1* | *AMP^R^  GST-slx9-1* | this study |
| pGEX-6P-1-*SSB1C* | *AMP^R^ GST-SSB1C* | Maurer *et al.*, 2001 |
| pGEX-2TEV-*YRB1* | *AMP^R^ GST-YRB1* | Schlenstedt *et al.*, 1995 |
| pQE30-*RIO2* | *AMP^R^ HIS_6_-RIO2* | this study |
| pQE70-*XPO1* | *AMP^R^ XPO1-HIS_6_* | Maurer *et al*., 2001 |
| pQE9-*GSP1* | *AMP^R^ HIS_6_-GSP1* | Maurer *et al.*, 2001 |
| pQE9-*GSP1Q71L* | *AMP^R^ HIS_6_-GSP1Q71L* | Maurer *et al.*, 2001 |
| pQE9-*GSP1Q71L^R142A K143A^* | *AMP^R^ HIS_6_-GSP1Q71L^R142A K143A^* | this study |
| pQE9-*GSP1Q71L^R142E K143E^* | *AMP^R^ HIS_6_-GSP1Q71L^R142E K143E^* | this study |
| pQE9-*GSP1∆CQ71L* | *AMP^R^ HIS _6_-GSP1∆CQ71L* | Maurer *et al*., 2001 |
| pRS313-*RIO2* | *AMP^R^ RIO2 HIS3 CEN* | this study |
| pRS313-*RIO2∆NES* | *AMP^R^* *RIO2∆NES HIS3 CEN* | this study |
| pRS313-*SLX9* | *AMP^R^* *SLX9* *HIS3 CEN* | this study |
| pRS313-*slx9-1* | *AMP^R^* *slx9-1* *HIS3 CEN* | this study |
| pRS313-*YRB2* | *AMP^R^* *YRB2 HIS3 CEN* | this study |
| pRS314-*MEX67* | *AMP^R^* *MEX67 TRP1 CEN* | this study |
| pRS314-*mex67∆loop* | *AMP^R^* *mex67∆loop TRP1 CEN* | Yao *et al.*, 2007 |
| pRS314-*MTR2* | *AMP^R^* *MTR2 TRP1 CEN* | this study |
| pRS314-*mtr2∆loop116-137* | *AMP^R^* *mtr2∆loop116-137 TRP1* | Yao *et al.*, 2007 |
| pRS315-*RIO2∆NES* | *AMP^R^* *RIO2∆NES LEU2* | this study |
| pRS315-*RIO2* | *AMP^R^ RIO2 LEU2* | this study |
| pRS315-*RIO2-NMD3NES∆1* | *AMP^R^ RIO2-NMD3NES∆1 LEU2* | this study |
| pRS315-*RIO2-NMD3NES* | *AMP^R^ RIO2-NMD3NES LEU2* | this study |
| pRS315-*RPS2-GFP* | *AMP^R^* *RPS2-GFP LEU2* | Altvater *et al*., 2012 |
| pRS315-*SLX9* | *AMP^R^* *SLX9 LEU2* | Faza *et al*., 2012 |
| pRS315-*slx9-1* | *AMP^R^* *slx9-1 LEU2* | this study |
| pRS315-*slx9-1-GFP* | *AMP^R^* *slx9-1 -GFP LEU2* | this study |
| pRS315-*SLX9-GFP* | *AMP^R^* *SLX9-GFP LEU2* | this study |
| pRS315-*YRB2* | *AMP^R^* *YRB2 LEU2* | this study |
| pRS316-*RPS2-GFP* | *AMP^R^* *RPS2-GFP URA3* | Milkereit *et al.*, 2003 |
| pRS316-*SLX9* | *AMP^R^* *SLX9 URA3* | Faza *et al*., 2012 |
| pRS316-*slx9-1* | *AMP^R^* *slx9-1 URA3* | this study |
